# Temporary severe bradycardia due to pacemaker programming

**DOI:** 10.1007/s12471-016-0854-2

**Published:** 2016-06-09

**Authors:** A. Böhm, R. G. Kiss, P. Bogyi, G. Z. Duray

**Affiliations:** Department of Cardiology, Military Hospital, Budapest, Hungary

## Answer

**Fig. 1:**
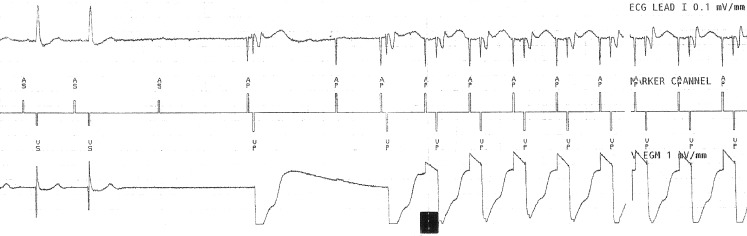
ECG recording during carotid massage: Lead I, marker channel, ventricular electrogram

As a result of carotid massage, sinus bradycardia and AV blockade develop, the A sense-A sense interval is lengthened to 1360 ms, and the P wave is not followed by R wave transmission. Since there is no further P wave, an atrial spike follows after 1200 ms, with the lower rate set at 50 bpm. Due to the managed ventricular pacing (MVP) function, at 80 ms following a non-transmitted atrial event, ventricular stimulation follows; this can be seen after the 2nd atrial paced beat (Ap) which is then followed by another Ap at 1200 ms, without a subsequent QRS, as a result of carotid sinus massage. The next Ap-Ap (3rd and 4th) interval is 600 ms (Fig. 1). This could be explained as follows: the rate drop response is activated by a rhythm of 50 bpm frequency for two beats, leading to an atrial-ventricular stimulation of 100 bpm. The pacemaker is working correctly; the interesting ECG recording is due to the combination of MVP and rate drop functions. Considering the significant decrease in ventricular frequency observed in this case, MVP and rate drop functions should not be programmed simultaneously.

